# Quantifying the web browser ecosystem

**DOI:** 10.1371/journal.pone.0179281

**Published:** 2017-06-23

**Authors:** Sela Ferdman, Einat Minkov, Ron Bekkerman, David Gefen

**Affiliations:** 1 Department of Computer Science, University of Haifa, Haifa, Israel; 2 Department of Information Systems, University of Haifa, Haifa, Israel; 3 Department of Information and Knowledge Management, University of Haifa, Haifa, Israel; 4 LeBow College of Business, Drexel University, Philadelphia, PA, United States of America; University of Cape Town, SOUTH AFRICA

## Abstract

Contrary to the assumption that web browsers are designed to support the user, an examination of a 900,000 distinct PCs shows that web browsers comprise a complex ecosystem with millions of *addons* collaborating and competing with each other. It is possible for addons to “sneak in” through third party installations or to get “kicked out” by their competitors without user involvement. This study examines that ecosystem quantitatively by constructing a large-scale graph with nodes corresponding to users, addons, and words (terms) that describe addon functionality. Analyzing addon interactions at user level using the Personalized PageRank (PPR) random walk measure shows that the graph demonstrates *ecological resilience*. Adapting the PPR model to analyzing the browser ecosystem at the level of addon manufacturer, the study shows that some addon companies are in *symbiosis* and others *clash* with each other as shown by analyzing the behavior of 18 prominent addon manufacturers. Results may herald insight on how other evolving internet ecosystems may behave, and suggest a methodology for measuring this behavior. Specifically, applying such a methodology could transform the addon market.

## Introduction

Web browsers have become a major component of the routine human-computer interaction, with some operating systems based entirely on browsers (e.g., ChromeOS by Google [1]). Browser extensions, also known as *addons*, are computer programs that (as the name suggests) extend, improve, and personalize browser capabilities. More than 750 million addons were downloaded and installed by Google Chrome browser users as of June 2012 [2]. Some examples of addons include an extension that allows visually impaired users to access the content of bar charts on the Web [3], an extension that addresses users’ security concerns by seamlessly producing a unique password for each website the user accesses [4].

Internet software companies are very interested in installing their addons, and particularly *toolbars*, on users’ machines. Toolbars are GUI widgets that typically reside in the upper part of the browser’s window, extending the browser’s functionality. Toolbars can collect information about the browsing history of the user (e.g., Yahoo! Toolbar [5]) and can redirect user search activity to a specific search portal (e.g. MyWebSearch.com). Crucially, the company that owns the search portal, and typically also the toolbar, receives payments from ad providers per user click on the ads it displays (primary ad providers are Google and Yahoo!). This revenue generation model is used extensively by software companies that distribute freeware products [6]. For example, 45% of AVG Antivirus Technologies sales in 2012 were generated by its browser toolbar [7]. It was estimated that Google, the biggest Web advertising firm, might have lost 1.3 billion in revenue in 2013 because of changes to its policy with respect to toolbars and a resulting shift of some addon distributors to Google’s competitors [8, 9].

Consequently, addons compete with each other over resources (such as battery, memory, disk space, and computing power) and user attention. Regardless of how intelligent they are, they may be aware of each other and may “piggyback” on each other or uninstall each other. Addon behavior within the Web browser is characterized by addons making their own decisions independently and often unbeknown to the user, which comprises a complex ecosystem with the user being just one of the participants. A key issue in understanding that ecosystem, responding to it, regulating it, and transforming it into a mature market is the current inability to show that it is inherently stable and measurable. This study addresses that issue.

More broadly, the Web browser ecosystem is characteristic of the types of systems discussed in the seminal paper by Russell et al. [10] that poses core questions about the legal, ethical, and structural regulation of decisions that can be made by intelligent systems that compose of both human and machine decision making. Past research into that arena looked into Human-Computer Interaction (e.g., [11]), mostly being concerned with how one human communicates with one machine, or how humans communicate with each other with the help of machines. Likewise, Multi-Agent Systems research (e.g., [12]) deals with cooperation between machines, while overly ignoring environments in which machines do not cooperate with each other, are not designed to do so, or are unaware of each other. In contrast, this paper deals with the wider ecosystem in which machines both compete and collaborate with each other.

Addressing such a dynamic ecosystem, this paper shows the applicability of a Personalized PageRank (PPR) random walk in the heterogeneous graph of users, addons, and addon description terms, to quantify the Web browser ecosystem. This could be a first step toward monitoring and regulating independent machine behavior. An example of independent machine behavior within the addon ecosystem is an antivirus tool that is being installed on a laptop: what should it do about another antivirus tool that was preinstalled on that laptop? Such questions are becoming more pertinent in the context of addons because—while browser extensions can be installed proactively—they are often “silently” installed on one’s machine by a third party, typically, as the user downloads some other program or installs a “software bundle”. We find that these questions addressed by this research are both of theoretical significance, as well as of much economic impact.

## Research questions and their addon ecosystem context

The Web browser ecosystem is a complex evolving one. Addons are installed and uninstalled on user machines. New addons introduced by software companies become prevalent or fade over time. New addon companies enter, and older players gain or lose power. Companies establish partnerships or compete with each other (and sometimes both). To mention but a few of its dynamic characteristics. These developments occur solely within the digital media—addons being software executables—with each addon having a lifecycle of events and a spectrum of interactions with its environment. All this happens on a daily basis and is mostly hidden from the user who may not even be aware of the vibrant “life” on his/her Web browser.

Addons are in a symbiotic relationship when at least one of them benefits from the other. For example, an addon may get installed on a user’s machine during (or following) the installation of another addon. This can be considered a direct benefit to the latter addon, as it would not have reached the machine if the other addon was not installed on it. Often, addons of the same company are installed in a bundle. In some cases, addons companies may even have a distribution agreement such that one company provides the means for installing the other company’s addons. Clashes occur when an addon “kicks out” other addons. There are a variety of reasons for a clash. A clash may happen, for example, when one company’s addon removes another company’s addon because the two companies’ products directly compete with each other. Of course, also the user (i.e. the computer owner) plays an important role in the addon ecosystem: some users “hunt down” and remove addons that occasionally appear in the computer’s browser; other users are more tolerant—they let addons live in the browser for a long time and do not mind more addons to be installed over time.

All these processes occur in the Web browser *habitat*. This habitat is observably *ecologically stable* (browsers do not crash frequently) and shows *resilience*: if not disturbed, the habitat will remain approximately the same, and if disturbed from outside then it will “remember” its stable state and try to recover.

Addressing the research objective of quantifying independent machine behavior in the context of addon ecosystems, the first research question aims to establish that the Web browser addon habitat can be verified as resilient. Building on that verification, the next research questions address the symbiosis and clash characteristics of that habitat.

**RQ1: Can Web browser habitat resilience be verified?**

**RQ2: Can the degree of addon symbiosis and clash be measured?**

The research questions are addressed by analyzing records of user-addon associations collected from anonymous users all over the world. The original data consisted of the list of addons detected per user, including their textual descriptions and installation paths. That data was cast into a relational graph in which typed nodes correspond to distinct *user*, *addon*, and *term* objects. In this representation a habitat observed on an individual user’s machine forms a star-shaped sub-graph in which a node corresponding to the *user* is linked to nodes corresponding to the *addons* that reside on that user’s machine. Those *addon* nodes may be further linked to lexical *terms*, derived from their textual descriptions. Multiple habitats can be connected in the joint graph. For example, each *addon* is directly connected to all the *users* that have it installed. The graph representation is compact, supporting efficient processing of large-scale data. Importantly, graph-theoretic methods can be employed to assess structural inter-node relatedness.

The ability to verify habitat resilience (RQ1) is measured by showing that if a random addon is removed from the habitat then, given the identity of the remaining addons in that habitat and inter-habitat relationships as registered on the relational graph, the missing addon can be identified. The significance of being able to do so is shown by verifying that a Personalized PageRank (PPR) random walk is better than a “one-fits-all” method such as the *popular choice* method. Given that habitat resilience can be verified, the subsequent RQ2 research question shows that two defining characteristics of a habitat, symbiosis and clash, can also be measured by assessing the relationships among addon companies. A graph-based measure of *relative importance* is employed for this purpose. The results suggest the possibility to monitor and regulate independent machine behavior. We claim that PPR may be a candidate algorithm for doing so, and show its ability to detect business alliances and rivalries in digital media.

## Related research

Gaining insight from biology to computer science is a topic of ongoing research [13]. Examples include the popular analogy of malicious software to viruses [14], the study of epidemic propagation in networks [15], the comparison of information dissemination on social networks to an evolutionary process [16], and more. This study follows in the footsteps of previous research that outlined an analogy between biological ecosystems and the collective behavior of players, or processes, in the software industry. While that literature, discussed next, is theoretical and anecdotal, this study reports empirical results using real-world data that shows characteristics of software ecosystems arguably similar to those of biological ecosystems. The next sections will define ecosystems in the context of previous research, and then survey research related to the methodology used in this study.

### Business and software ecosystems

It has long been suggested that companies should not be viewed as individual entities, but rather as part of a *business ecosystem* [17, 18]. Applying this paradigm, companies might be thought of as corresponding to species in a biological ecosystem. Like its biological counterpart, a business ecosystem is assumed to gradually develop from a collection of elements to a structured community, and, likewise, each member of a business ecosystem ultimately shares the fate of the network as a whole, regardless of its relative strength.

To put this study into perspective, we overview recent research focused on *software ecosystems* [19–22], studying the complex relationships among companies in the software industry. Manikas and Hansen [21] defined a software ecosystem as the interaction of a set of actors on top of a common technological platform that results in a number of software solutions or services. As an example, they considered the iOS ecosystem in which *Apple* provides a platform for selling applications in return for a yearly fee and 30% of revenues of application sale. According to Manikas and Hansen, software ecosystems are characterized with a wide spectrum of symbiotic relationships: two actors might have mutual benefits, be in direct competition (antagonism), be unaffected (neutralism), or be in a position where one company is unaffected while the other is benefiting (amensalism) or harmed (parasitism) by their relationship. Manikas and Hansen noted that little research had been done in the context of real-world ecosystems. Other researchers used the term “software ecosystems” to describe more technical aspects concerning the development of software systems that involves multiple players and must adapt to new environment or requirements [23, 24].

To the best of our knowledge, the current work is the first that studies interactions between players in the Web browsers addons domain. This Web browser ecosystem differs from organization-centric software ecosystems previously studied in the literature (e.g., [25]) where an organization develops a software ecosystem around its offering, such as in the case of *Salesforce* that created a marketplace of third-party extensions to its products [26]. In the Web browser ecosystem there is no organization that can regulate addon behavior. Moreover, browser addons can interact directly with each other, even removing each other from the user’s machine, which is not allowed in a the regulated ecosystem of an organization. Jansen and Cusumano [26] found that a significant difference between the software and ecological ecosystems is that software species can “consciously” decide to exit the ecosystem as opposed to species in a biological ecosystem. That distinction, however, may not readily apply to the browser addon ecosystem because addons do not leave the system at their own will—once installed, only external factors limit their survival.

The resilience of a biological ecosystem is defined as the amount of disturbance that it can withstand without changing its self-organized processes and structures [27], or as the time required for the ecosystem to return to its stable state after a perturbation [28]. Dhungana et. al. [20] define a sustainable software ecosystem as one that can survive a significant habitat changes coming from competitors. Along the same lines, this study defines ecological resilience as the ability of a Web browser ecosystem that is artificially disturbed by extracting an existing addon to “remember” its original state to the extent that the missing addon can be predicted. Such *ecological memory* is a main component of ecological resilience, playing a major role in reorganization of ecosystems [29]. Ecological memory includes the biological legacies within habitat and the genetic composition of populations. As described by Schaefer [30], ecological memory is encapsulated in soil properties, spores, seeds, stem fragments, species, populations and other remnants that influence the composition of the replacement ecosystem and may also support ecological restoration. In particular, an internal component of ecological memory consists of remnants of species in the immediate area and an external component consists of the surrounding areas. The internal component of the addon ecosystem studied in this research corresponds to the addons installed at the habitat of an individual user, and the surrounding areas–to the objects that directly connect with the user’s environment in the global graph.

### Graph-based data representation

The definitions above imply that an ecosystem can be presented as a set of objects that interact in various ways among themselves, and possibly with other environmental objects. Such relational schema is naturally represented using a heterogeneous typed graph in which nodes denote entities and edges denote inter-entity relationships [31, 32]. A plethora of well-studied and efficient methods exists that can identify global phenomena in such a graph and evaluate the relatedness between remote entity pairs [33, 34]. Nonetheless, only few studies analyzed ecosystems using graph-based quantifiable measures. One such study was conducted by Blincoe et. al. [24] who aimed at identifying ecosystems among software projects developed in the *GitHub* platform [35]. Blincoe et. al. constructed a graph in which vertices denoted software projects on *GitHub* and edges represented technical cross-project references. Multi-project ecosystems in their graph were then identified using a community detection method and displayed visually. This study takes graphing ecosystems a step further. The current study uses quantifiable graph measures to establish that addons form an ecosystem that is resilient and then to detect collaboration and adversary relations between the members of the ecosystem.

To establish that addons form an ecosystem that is resilient, resilience is formalized as a *link prediction* problem. The general task of link prediction aims at estimating whether a link should exist between two disconnected nodes in a graph based on the graph’s structure [32, 36–38]. Link prediction is often used for recommendation purposes such as in online social networks where it is applied to identify likely but “missing” positive links that can then be recommended as promising friendships [39] and such as automatic enrichment of knowledge bases that are represented as a relational graph with missing edges [40]. Often, link prediction is evaluated by removing known existing edges, and evaluating the extent to which these edges can be recovered based on the remaining graph.

The current study utilizes that ability to address RQ1 using the PageRank method [41, 42] and its Personalized PageRank (PPR) variant (sometimes referred to as random walks with restart (RWR), see [43]). The well-known PageRank model applies a random walk process where at each step a random walker stochastically chooses to either traverse an outgoing link or to jump (“restart”) to a random node in the graph. This random walk process converges to a stationary node probability distribution in which the scores of the nodes represent their structural centrality in the graph. The main drawback of the PageRank model is that it fails to incorporate node-specific context. The *Personalized* PageRank method addresses this shortcoming by applying a minor enhancement: rather than “jumping” to some node uniformly at random, the restart operation is confined to a distribution of interest which is referred to as a *query*. In such a setting the PPR score of a given node reflects its relevance with respect to the query.

The Personalized PageRank random walk metric has been applied to a large variety of tasks, including ranking Web pages and influential social media users with respect to topics of interest [44, 45] and personalized and context-sensitive item recommendation [46]. In addition to Web networks and social media, PPR has been successfully applied to other domains, including personal information management [31], computational linguistics [47], and computational biology [48].

A few previous studies attempted to automatically identify competition relationships between companies that offer similar products and thus compete over market share. Most existing studies used text documents as their main information source (e.g., [49, 50]). In the context of the current study, collaboration (or *symbiosis*) is defined as co-existence in the same habitat (same user browsers) while competition (or *clash*) as addons eliminating each other. Notably, the graph contains no explicit indication on positive or negative relationships between nodes so existing methods (e.g., [51, 52]) cannot be applied to infer symbiosis from positive links and clashes from negative links. Instead, those relationships must be uncovered solely based on the graph structure in an unsupervised manner.

## Data

**Fig 1:**
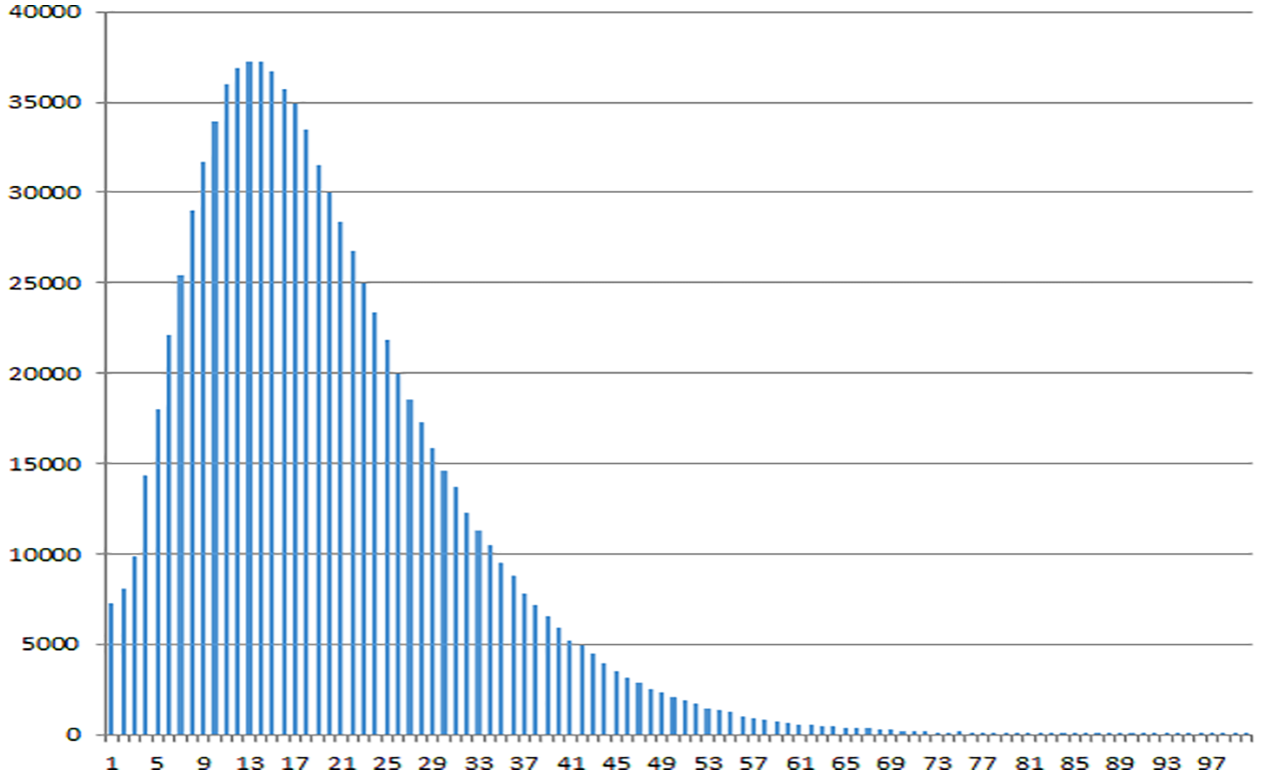
Histogram of the number of users over the number of addons they have on their machines.

The current study examined large-scale authentic data describing browser addons installed on real users’ computers. These data were collected from users all over the world who agreed to anonymously share this information. It is a common scenario that users maintain multiple browsers. For example, *Microsoft Internet Explorer* is pre-installed on Windows machines, and many users install an additional browser. The database lists addons installed on multiple browsers, including *Microsoft Internet Explorer*, *Mozilla Firefox*, and *Google Chrome*. The data were stored using a relational database on the cloud at *Amazon* RDS. As of 2013, the dataset included over 1.5 billion records. For the purpose of this study, a subset of the data was considered. That subset included all of the records collected over a period of two months between August 1, 2013 and October 1, 2013. For every user there could be multiple records collected, describing a snapshot of his/her machine on a daily basis. As the length and frequency of data collection were inconsistent over time and across users, only the records collected at the earliest date per user were considered. Overall, the dataset contains 17,942,715 user-addon associations that correspond to 907,844 distinct users and 256,458 distinct addon descriptions. Fig 1 shows the distribution of the number of addons installed per user machine. As shown, most users had between 9 and 21 addons on their machines.

The data about each addon consisted of:

- **Addon type.** These form a closed set, where prevalent values are ‘extension’, ‘toolbar’, or ‘BHO’ (Browser Helper Object, an Internet Explorer addon).
- **File name.** This includes the full path at which the addon software is installed on the user machine.
- **Name.** Addon’s name.
- **Description.** A textual description of the addon’s functionality.

**Fig 2:**
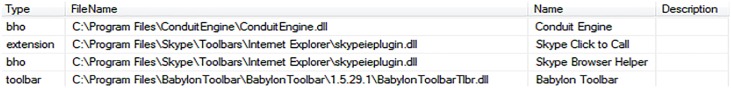
User addons record: Sample user 1.

**Fig 3:**
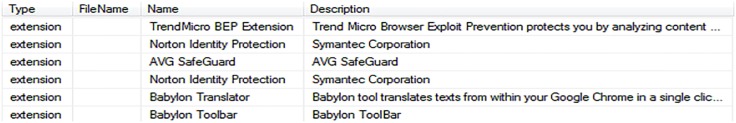
User addons record: Sample user 2.

Figs 2 and 3 show two addon records associated with two different users. The information specified is browser-dependent and sometimes missing. In these two cases, addon descriptions are missing for the first user and the path information is missing for the second. For each user–addon pair at least one attribute (path, name, or description) is guaranteed to be present in the data.

**Fig 4:**
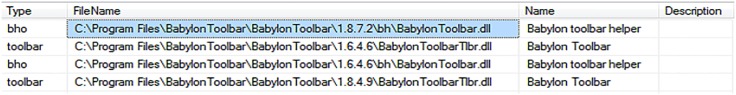
Example of coreferent addon records with different software version numbers.

Importantly, similar addon software may be described by multiple different records, i.e., the addon records lack normalization. Fig 4 illustrates this variability across records. Sources of variance include different installation paths, availability or absence of attribute values, and different software version numbers (e.g., 1.8.7.2 vs. 1.6.4.6 in Fig 4). Furthermore, the user base is international and is therefore multilingual. The database includes no tracking of user’s or other programs’ actions. It was therefore impossible to determine which party initiated the installation (or removal) of an addon.

### Graph representation

The dataset corresponding to the graph consisted of over 1.3 million nodes and over 18.5 million edges. Detailed statistics are provided in Table 1. The data were represented using a relational graph schema. Each node in the graph represents a unique object that belongs to one of the following node types:

1. **User.** An individual user is represented as a graph node that carries his/her unique user id.
2. **Addon.** These nodes correspond to specific addons, defined as the concatenation of all of the addon’s attributes; namely, file path, addon name, and description. Addon names often include full file-system path information such as “C:/Program Files (x86)/Skype/skype1.dll”. To avoid registering an addon twice solely due to minor discrepancies in the installation process path prefixes, such as “C://Program Files (x86)/skype”, were removed. Additionally, addons with slightly different names, such as different version numbers, were unified by the random walk. This was done by splitting addon names into tokens and linking the respective *addon* and *term* nodes to maintain connectivity between multiple versions of the same *addon*.
3. **Term.** The text strings that comprise addon names was parsed into individual terms, represented as graph nodes, as illustrated below.

**Table 1:** Graph statistics.

Type	Quantity
*All nodes*	1,331,814
*User nodes*	907,844
*Addon nodes*	256,458
*Term nodes*	167,512
*High degree nodes* (>500)	2,430
*All edges*	18,552,622
*User* − *addon edges*	17,612,159
*Addon* − *term edges*	940,463

**Fig 5:**
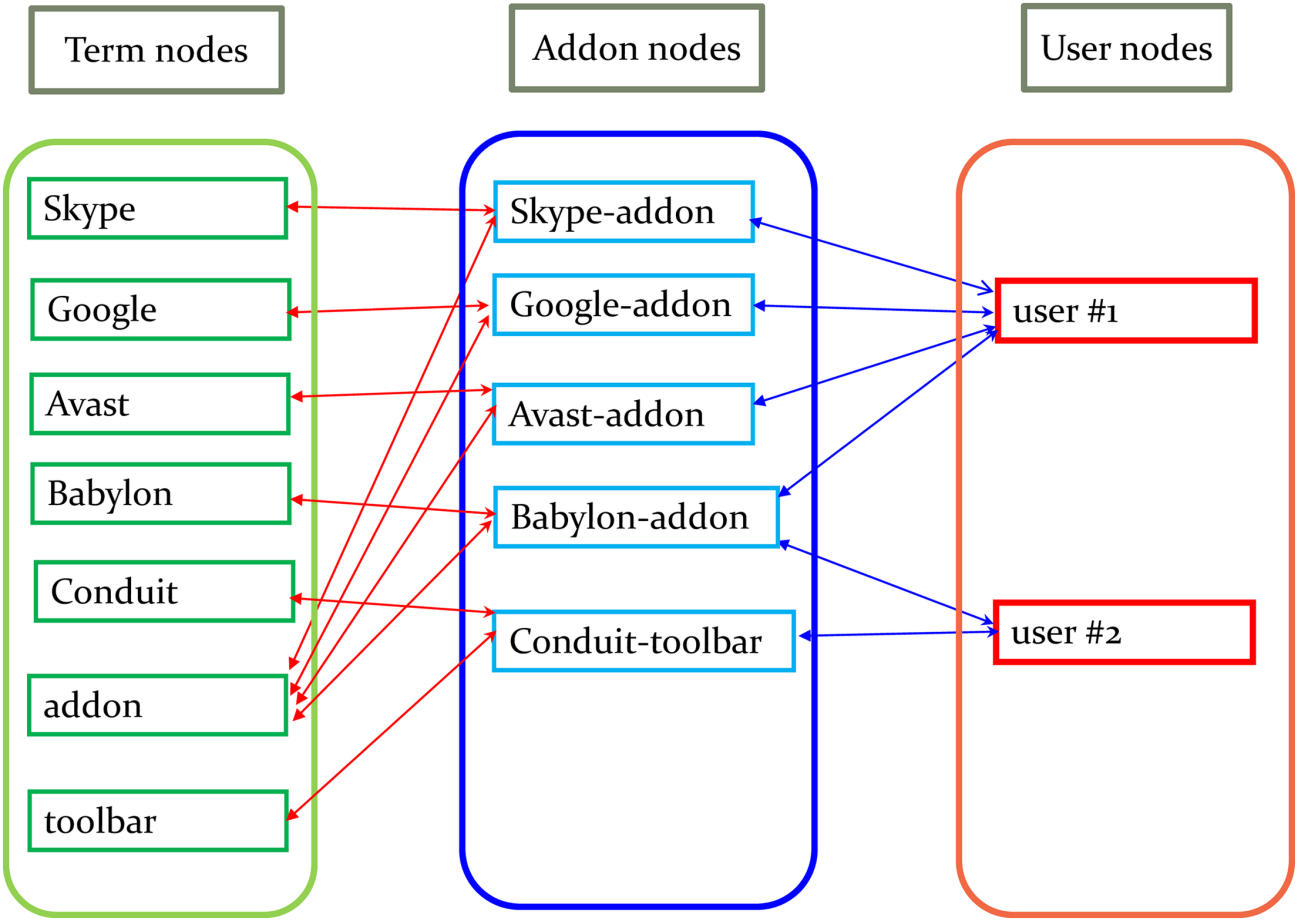
User-addon-term connections.

There are two types of edges in the graph. The first type represents the structural association between each *user* and each *addon* installed on his/her machine. The second type links each *addon* node to all *term* nodes that comprise its Bag-of-Terms representation. Inverse edges exist between every connected node pair so the graph may be viewed as undirected. Fig 5 illustrates the graph structure. A *user* is represented as a graph node that is connected to all its corresponding *addon* nodes with undirected edges. Each *addon* node, in turn, is connected to all its *term* nodes. In the specific example of Fig 5, *user 2* has two addons (*Babylon-addon* and *Conduit-toolbar*) that are connected to their term nodes (*Babylon*, *Conduit*, *addon* and *toolbar*). To construct the graph, the algorithm iterated over all the users in the dataset to create user nodes. For each user, it then iterated over all his/her addons, and mapped each addon to a unique node. Finally, each *addon* node was tokenized and lower-cased into single words, and each unique word mapped to a respective *term* node.

**Fig 6:**
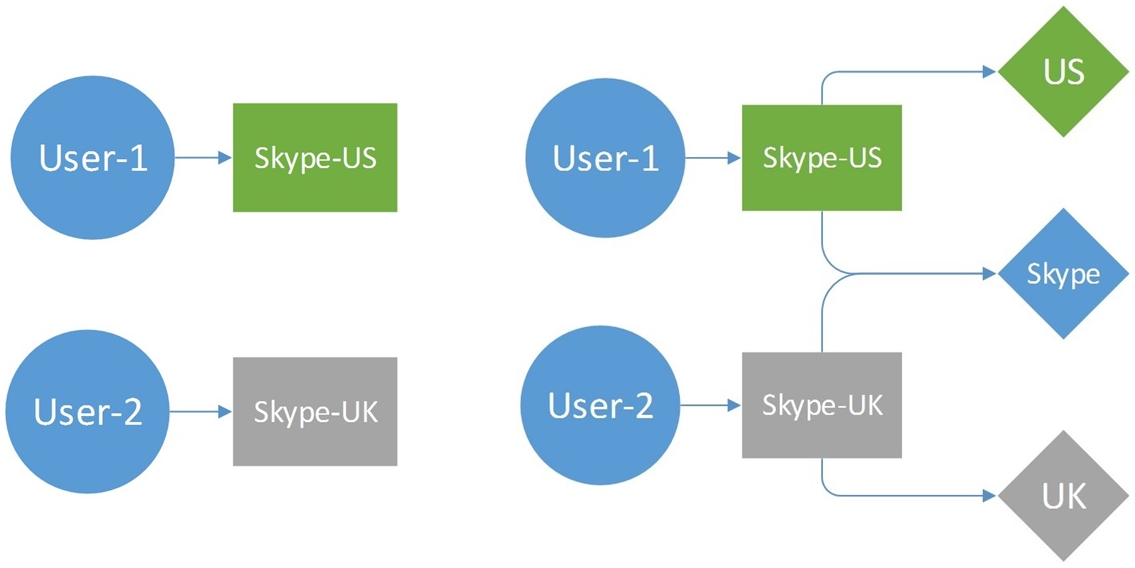
Example of the constructed graph, without the term layer (left) and with the term layer (right).

Besides being compact, the graph representation is advantageous in that similar entities reside in high proximity to each other. Consider, for example, two *addons* “Skype-US” and “Skype-UK” that have non-identical names, but share the term “Skype” which indicates in this case that they are variants of the same addon. Fig 6 shows how term nodes help construct a connected graph where similar nodes are close to each other. Two disconnected segments on the left panel get connected to each other through the “Skype” term node, which leads to a close relatedness between *User 1* and *User 2*.

## Assessing research question 1. Can web browser habitat resilience be verified?

Our link prediction experiment for assessing the resilience of a browser habitat was designed as follows. A direct link between a *user* and an *addon* was randomly removed from the graph, and the identity of this “missing” addon was then predicted based on information about remaining *addon* members at that user’s environment, applying PPR to rank the *addons* by their graph-based association with the user’s environment. The objective of the experiment was to show that PPR could produce better results than an algorithm that ignored ecological memory, and did not model the user’s environment.

More formally, let *U* denote the set of users represented as nodes in the underlying graph *G*. Every individual user *u* ∈ *U* is linked in *G* to the set of addons installed on *u*’s machine, *A*(*u*). Having disconnected the link between a random user *u*_*i*_ and an addon *a*_*j*_ ∈ *A*(*u*_*i*_), we wish to evaluate the extent to which the missing link between *u*_*i*_*a*_*j*_ can be recovered based on the remaining information about the user’s environment *A*(*u*_*i*_)′ = *A*(*u*_*i*_)∖{*a*_*j*_} and *G*. Using information retrieval terminology, in what follows we will refer to *A*(*u*_*i*_)′ as a *query*. Candidate responses in this case are all *addons* that are not known to be associated with the user, i.e., *A*∖*A*(*u*_*i*_)′, having this candidate set include the target response *a*_*j*_. These candidate nodes are ranked by their estimated relevance to the query. Accordingly, performance is evaluated quantitatively with respect to the rank of the ‘missing’ *addon* *a*_*j*_ across multiple instantiated queries.

The experiment employed PPR [41] to compute query-specific relevance scores based on the link structure of the graph. An overview of the PPR algorithm can be found in [53]. In brief, PPR follows the well-known PageRank algorithm that was originally designed to assign a “centrality” score to webpages. PageRank models the behavior of a random surfer who at any given time may choose to either follow a hyperlink to another webpage or to jump (“reset”) to some random page on the Web. The probability distribution of finding the surfer at any of the graph nodes at time step *d* is computed iteratively, as follows:

$$\begin{array}{c} V_{d+1}=\left(1-\alpha \right){\left[\frac{1}{N}\right]}_{1\times N}+\alpha MV_{d} \end{array}$$ (1)

where the total number of nodes (webpages) is *N*, and *M* is a transition matrix that models the probability that the surfer moves to page *j* from page *i* following a hyperlink. The probability that the surfer chooses to proceed by following some hyperlink is *α*, and the probability of the alternative action (resetting the walk) is (1 − *α*). The distribution *V*_*d*_ is guaranteed to converge to a unique stationary distribution V* in which node scores designate the respective documents’ centrality in the network [41].

The PageRank centrality scores reflect the entire network’s structure. The *Personalized PageRank* variant adjusts the random walk model to generate node rankings considering the specific preferences of user *u*. Accordingly, the random walk scheme is modified as follows:

$$\begin{array}{c} V_{d+1}=\left(1-\alpha \right)V_{u}+\alpha MV_{d} \end{array}$$ (2)

where *V*_*u*_ (the *query*) denotes a distribution over nodes that are of interest to user *u*. PPR scores, derived from the corresponding stationary state distribution, reveal structural similarity, or relevance, of graph nodes with respect to the query nodes. PPR scores for some graph node *z* and any single query node *x* equal a summation over all the connecting paths between *x* and *z* (including cyclic paths, and paths that cross *z* multiple times) where paths are weighted by their traversal probability [53, 54]. In other words, the graph walk distributes probability mass from the start distribution *V*_*u*_ through edges in the graph—incidentally accumulating evidence of similarity over multiple connecting paths. Due to the reset operation, having a fixed fraction of probability mass reassigned to the query nodes at each step, the weights of the paths between *x* and *z* exponentially decay as their length increases. This implies that graph nodes that can be reached from the query nodes over shorter connecting paths, as well as over multiple connecting paths, are considered more “important” with respect to the query.

### Predicting the missing addon using PPR

Applying PPR the current study sets *V*_*u*_ to be uniform over *A*(*u*)′ and zero otherwise. *M* assumes equal importance to all of the graph edges. That is, the transition probability from node *i* to a linked node *j* is defined as: $M_{ij}=\frac{1}{|N_{i}|}$, where *N*_*i*_ is the set of nodes linked over an outgoing edge from *i*. For example, suppose that an addon node *i* is linked to two *term* nodes and five *user* nodes then the probability of reaching any of these nodes using the transition operation equals $\frac{1}{7}$. It is generally possible to assign varying edge weights, either parametrically according to edge types, or deriving such weights from local edge properties. Previous works also considered learning a selective set of meaningful paths in the graph that link a query with target nodes [31, 40]. The stationary distribution of the random walk process manifests long-range relationships in the graph. The resulting computed PPR vector assigns a score to every node in the graph. Based on those scores, the ranking of *addon* nodes is be generated.

### Experimental setup and evaluation

To build the test data, a set of labeled queries was derived from the data. Each query corresponded to a randomly selected user *u*. Once the user node *u* is selected, one of its linked *addons*, *a*, was randomly selected and then removed from the user’s “profile” by removing the link between the nodes *u* and *a* from the graph. The process is outlined below. The PPR query is set to be the rest of user *u*’s addons. PPR was run on the entire graph with respect to the constructed query.

1. Pick uniformly at random a *user* node *u* from the graph.
2. Select the set of all *addon* nodes linked to *u*, *A*_*u*_.
3. Pick uniformly at random an addon node *a* from the set *A*_*u*_.
4. Remove the edge between nodes *a* and *u* in the graph.
5. Let the query *V*_*u*_ be a uniform distribution over *A*_*u*_∖{*a*}, and the correct response to the query (the *label*) be *a*.

Performance was assessed using metrics adapted to the evaluation of ranked lists. Note that in our settings, there is a single known “correct” answer, i.e. the missing addon. The evaluation metrics assess the extent to which the correct answer is included at the top of the ranked list of addons as constructed by the PPR, across the set of test queries. Obviously, user and term nodes, as well as the query addon nodes, are discarded from the evaluated ranked list. The evaluation process applied the following measures:

1. **Recall at rank *k*.** This is the fraction of queries in which the relevant response is included among the top *k* ranks (see also [31]). Concretely, the non-interpolated recall at rank *k* of a given ranked list is defined to be 0 for each rank *k* = 0, …, *k*_*i*−*i*_, where *k*_*i*_ is the rank that holds the single correct entry, and 1 for ranks *k* ≥ *k*_*i*_. The (mean) recall at rank *k* averages the recall scores at each rank *k* across the rankings of multiple queries. Thus, mean recall is in the range [0, 1] at each rank *k*. For example, if recall at rank 3 is 0.7, this means that for 70% of the queries the correct answer appears among the top 3 ranks of the generated ranked lists.
2. **Mean Reciprocal Rank (MRR).** The mean reciprocal rank metric [55] considers the position of the (only) correct answer in the ranked list. The non-interpolated reciprocal rank is the inverse of the position of the correct item (addon) in the ranked list for a given query. The MRR score is the mean reciprocal rank across all of the queries: $MRR=\frac{1}{Q}\sum_{q=1}^{Q}\frac{1}{rank_{q}}$. Unlike the recall-at-rank measure that ignores the results below rank *k* for evaluation purposes, MRR is based on the full list of ranked items.

To increase robustness, the above measures were applied to evaluate query sets that consisted of 1000 labeled examples of randomly sampled user-addon pairs per experiment. Each experiment was repeated 4 times. The mean of the 4 evaluation scores per query set is reported together with the standard deviation. All the experiments were run on a fast and memory-efficient implementation of PPR included in *igraph* [56], a software library optimized for the processing large-scale graphs. The experiments were run on a standard PC using the 64-bit version of *igraph*. The entire graph was loaded into memory. A batch of 1000 PPR runs was completed within a few hours. In the experiments, we set the reset probability parameter *α* = 0.85 following [57].

### Results: Evaluating the effect of ecological memory

To assess the extent to which information about the remaining addons in a user’s machine is helpful for predicting the identity of a “missing” addon, the PPR results were compared with two non-personalized alternative ranking methods: ranking *addons* by popularity and by their (non-personalized) PageRank scores.

1. **Popularity baseline (POP).** This algorithm predicts the “missing” addon by ranking known *addon* items by their *popularity* which is determined by the total number of users associated with that addon.
2. **PageRank baseline (PR).** This algorithm computes for each addon its non-personalized PageRank score in the underlying graph. The PageRank scores reflect the structural centrality of *addon* nodes in the graph.

**Fig 7:**
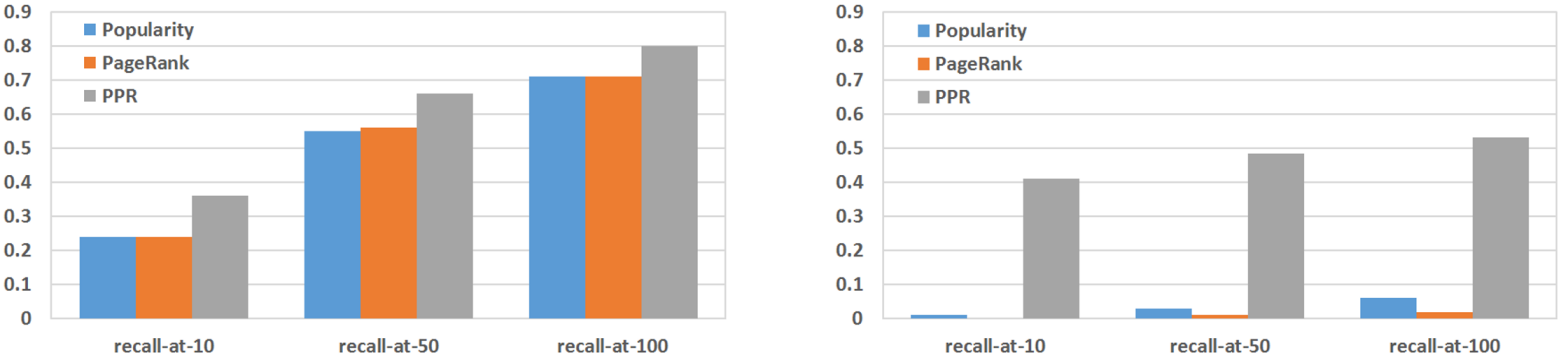
Left: recall at top ranks for the full graph, all the data. Right: recall at top ranks for the full graph, excluding the most popular addons.

According to both of these non-personalized approach, all queries are presented with the same addon ranked lists (excluding the specific query addons). Table 2 shows the results of the experiment for two graph variants, with and without *terms* layer. The best results per configuration are marked in boldface in the table. Running t-tests shows that the means of PPR are significantly higher (*p* < .0001) than POP and than PR in all the rows and columns in the upper half of Table 2. Fig 7 shows recall-at-*k* results using PPR compared with ranking-by-popularity and by PageRank scores, demonstrating the relative performance of the algorithms.

**Table 2:** Recall and MRR results averaged over 4 independent runs (standard deviations are in parentheses).

		Recall-at-10	Recall-at-50	Recall-at-100	MRR
		WITH POPULAR NODES			
*With terms layer* (Fig 6 right)	*POP*	0.243 (0.007)	0.555 (0.017)	0.711 (0.012)	0.104 (0.004)
	*PR*	0.243 (0.007)	0.559 (0.016)	0.711 (0.012)	0.104 (0.004)
	*PPR*	**0.354** (0.011)	**0.660** (0.009)	**0.801** (0.004)	**0.151** (0.006)
*Without terms layer* (Fig 6 left)	*POP*	0.240 (0.009)	0.553 (0.012)	0.719 (0.007)	0.101 (0.005)
	*PR*	0.240 (0.009)	0.563 (0.014)	0.719 (0.007)	0.101 (0.005)
	*PPR*	**0.350** (0.014)	**0.665** (0.008)	**0.809** (0.014)	**0.146** (0.008)
		WITHOUT POPULAR NODES			
*With terms layer* (Fig 6 right)	*POP*	0.006 (0.004)	0.034 (0.009)	0.065 (0.009)	0.004 (0.001)
	*PR*	0.001 (0.000)	0.009 (0.009)	0.022 (0.003)	0.001 (0.000)
	*PPR*	**0.405** (0.007)	**0.491** (0.007)	**0.527** (0.004)	**0.320** (0.005)
*Without terms layer* (Fig 6 left)	*POP*	0.000 (0.000)	0.000 (0.000)	0.000 (0.000)	0.000 (0.000)
	*PR*	0.001 (0.002)	0.012 (0.007)	0.027 (0.002)	0.001 (0.000)
	*PPR*	**0.401** (0.027)	**0.483** (0.027)	**0.521** (0.022)	**0.322** (0.027)

The lower half of Table 2 and the right hand side of Fig 7 show the results of a similar experiment over a graph variant in which high-degree nodes were removed. Those were defined as nodes with out-degree equal or greater than 500. This additional analysis was run because previous studies indicated that PageRank exhibits some bias in favor of high-degree nodes [58, 59]. Other studies indicated that the removal of high-degree nodes from an undirected power-law graph leads to a small approximation error, while improving the computational cost of the random walk [60]. In these additional experiments the performance of POP plummets as the popular addons are removed from the graph and from the sampled test queries: recall-at-10 is nearly zero (0.006) and recall-at-100 is also very low (0.065). PR results are even lower. In contrast, PPR remains effective: recall-at-rank-10 is 0.405 using PPR, reaching 0.491 and 0.527 at ranks 50 and 100, respectively. (Again, running t-tests shows that the means of PPR are significantly higher (p<.0001) in comparison with POP and in comparison with PR.) This indicates that popular nodes, which tend to occupy the top ranks, indeed “push” relevant yet less popular nodes to lower positions in the ranked lists—this phenomena is especially dominant in the one-fits-all “non-personalised” ranking approaches.

In conclusion, the personalized PPR produced significantly better results than non-personalized methods. This revealed structural association between the addons installed on a user’s PC is strong enough to enable recovering the identity of an addon that was deliberately removed. The Web browser ecosystem is resilient in this respect, answering in the affirmative RQ1. The next section, addressing RQ2, will now look into one possible reason for that. Namely, that some addons are complimentary or in competition with each other. Such *symbiosis* and *clash*, respectively, might possibly be due to business alliances and rivalries.

## Assessing research question 2. Measuring symbiosis and clash through PPR

*Symbiosis* in a Web browser habitat often occurs when addons of some companies are distributed via third parties. In such a process an addon’s installation is offered to a user as a part of some other product installation process. For example, a user installing *Skype* may by suggested to install also *Skype*’s “Click to Call” addon in all browsers. Another example: at the time the data for this section was collected, *Ask Toolbar* installation was integrated with Java installation so that, during the installation of Java, users were prompted to download and install also *Ask Toolbar* [61]. A *clash* effect can be observed when addons of one company are removed when addons of another company are installed on the same machine or if addons are not installed at all when another company’s addons are pre-installed on that machine. For example, *Kaspersky AntiVirus*, which develops addons for all browsers, treats *iMesh* addons as threats and removes them from the computer [62].

Needless to say, the life cycle of the addon ecosystem is mostly obscure for an outside observer. While some symbiotic effects may be visible to users (e.g. an addon is prompted to be installed during an installation process of another addon or a software product), some other symbiotic effects are hidden (e.g. undisclosed agreements between addon distributors). Clash effects, on the other hand, are almost always invisible. Besides a few well known conflicts between competing addon distributors that were widely covered in mass media [63], such competition is mostly invisible.

In order to identify symbiosis and clash relationship among addons, eighteen prominent addon distributors were manually chosen, detailed in Table 3. These companies are among the best known addons and toolbars distributors. Seven of the eighteen companies are antivirus and anti-malware companies. Although antivirus and anti-malware software aim to prevent unintentional addon installation, some antivirus companies are not only fighting unintentional addon installations but also distributing their own addons and toolbars. For example, *AVG Antivirus* distributes the *AVG Security Toolbar* which is detected by *Avast Antivirus* as malware. Indeed, in 2013 *Avast* *Antivirus* identified over 3.3 million different browser extensions for the three major browsers and published a list of the top ten companies whose addons were subject to removal [64]. Their updated list, published in 2015, did not change dramatically. Many of those companies are included in Table 3. In a blog post of July 9 2015, *Avast Antivirus* described the addon environment of a user’s Web browser, much as this study does, as an ecosystem where “addons fight against each other” [65]. Based on *Avast Antivirus* statistics on the forced removals of competing toolbars, some companies in Table 3 are among the top ten offenders. For example, *Conduit* performed more than 13 million removals of their competitors’ toolbars, *ASK* removed 11 million toolbars—and other companies were not far behind. *Avast Antivirus* itself has been accused of doing the same: “Avast is contradicting itself. Their latest product offers a built-in feature to rid your browser of toolbars, while offering a toolbar when installing their software.” [66].

**Table 3:** The list of addon distributors.

Company	Example Product	Description
*ASK*	Ask Toolbar	Advertisement/Search company
*AVG*	AVG Safe Search addon	AntiVirus/Advertisement company
*Avira*	Avira Browser Safety	AntiVirus company
*Babylon*	Babylon Toolbar	Advertisement/Search company
*Blekko*	Blekko Toolbar	Advertisement/Search company
*Conduit*	Conduit Toolbar	Toolbar provider company
*Google*	Google Toolbar	Advertisement/Search company
*Hotspot Shield*	Hotspot Shield VPN	Security company
*iMesh*	iMesh Search	Advertisement/Search company
*Incredimail*	MyStart by Incredimail	Advertisement company
*Kaspersky*	Kaspersky Protection Plugin	AntiVirus company
*Montiera*	Montiera Toolbar	Toolbar provider company
*Norton*	Norton Toolbar	AntiVirus company
*Softonic*	Softonic Web Search	Advertisement company
*SpeedBit*	Video Accelerator	Software company
*SweetIM*	SweetIM Toolbar	Advertisement/Search company
*Trend Micro*	Trend Micro Toolbar	AntiVirus company
*Zone Alarm*	Zone Alarm Toolbar	AntiVirus company

### Experimental design

To address RQ2, it was first necessary to identify the addon manufacturing company of each *addon*. An addon company often distributes hundreds or even thousands of addons. For example, *Kaspersky URL Advisor Firefox addon* and *Kaspersky Protection Chrome extension* are developed by the same company. Where possible, company name was identified within the addon installation path, name, or description. The default path of an addon package installation often contains the company’s name. And so, if a user does not change the default option, the company name will most probably be included in the addon’s path. For example, the addon path “C:\Program Files (x86)\Kaspersky Lab\Kaspersky Internet Security 2012\avp.dll” and its description “Kaspersky Protection extension” clearly show that the addon belongs to *Kaspersky*.

Having run that initial manual classification, PPR was applied to the original user, addon, and term graph to identify other addons that belong to one of the companies from Table 3 but were missed by the process in the previous paragraph. The procedure worked as follows. For each company in Table 3, a PPR query was constructed to contain the set of addons that were identified as belonging to that company. We expected an addon that belongs to that company but was not included in the query to be ranked higher relative to its original rank in the (non-personalized) PageRank. In other words, an addon that is ranked close to a set of addons that is known to belong to a certain company is a candidate to be an addon of that company even if its name, path, or description do not contain that company name. In this process, a non-personalized PageRank was first run on the entire graph; this provided a baseline position for each addon. That being done, a PPR was run for each company to identify addons that substantially improved their position in the ranked list. For example, if an addon was ranked of 100 in the non-personalized PageRank but was ranked 10 in PPR, that addon was manually examined to verify if it indeed belongs to that company.

The above procedure was performed iteratively. After a new addon-to-company relationship was identified, that relationship was added to the query and the PPR was rerun on that extended query. This iterative process continued until no more addons dramatically changed their rank. In practice, two iterations were enough for the process to converge. This process identified 24 additional addons as associated with the target companies. A manual check revealed that all those 24 addons were correctly identified. An example of an addon that drastically changed its rank is the addon *tbmyba.dll* which jumped from being ranked 1,200 to being ranked 15 after running PPR with *Babylon* addons in the query. Indeed, *tbmyba.dll* belongs to *Babylon* [67].

Having linked the addons to their respective companies, the symbiosis and clash between addon companies in RQ2 could be assessed. This assessment was done by constructing a set of PPR queries, one for each company, which contained all addons of that company. The PPR output for a target company *c*_*i*_ is a ranking of all the addons in the graph that reflects their association strength to *c*_*i*_. Addons of another company *c*_*j*_ that are ranked high in that PPR result compared to their ranking in non-personalized PageRank might suggest that the two companies have been engaged in a partnership, a *symbiosis*. Likewise, addons of a company *c*_*j*_ that are ranked considerably lower in the PPR ranking computed for company *c*_*i*_ as query compared to their position in non-personalized PageRank might suggest that the two companies *clash* with each other.

### Symbiotic relationships as addon set overlaps

If many addons of company *c*_*i*_ are installed on the same machines where addons of company *c*_*j*_ are also installed, it may be concluded that the two companies live in symbiosis with each other. This symbiosis can be measured with a Jaccard index [68]. The Jaccard index measures an overlap of two sets. We denote **M**_*i*_ the set of machines on which addons of company *c*_*i*_ are installed. Analogously, we denote **M**_*j*_ the set of machines on which addons of company *c*_*j*_ are installed. Jaccard index is then defined as:

$$\begin{array}{c} Jaccard\left(c_{i},c_{j}\right)=\frac{|M_{i}\cap M_{j}|}{|M_{i}\left|+\right|M_{j}\left|-\right|M_{i}\cap M_{j}|}, \end{array}$$ (3)

where the absolute value symbol means cardinality of a set. The matrix of Jaccard indices of the 18 companies is shown in Table 4. The few highlighted cells show the highest Jaccard indices. By analyzing the highlighted results, we can infer that Jaccard indices may not be a useful way to reveal relationships between addon manufacturers—simply because larger companies show larger overlaps. Indeed, companies such as *ASK*, *Babylon*, *Google*, and *Speedbit* have their addons installed on many machines in our dataset. No wonder that those addons happen to be installed together on the same machines. There is no evidence of symbiotic relationships between those companies—on the contrary, they are competitors.

**Table 4:** Jaccard index between addon distributors.

	asktoolbar	avg	avira	babylon	blekko	conduit	google toolbar	imesh	incredimail	hotspot	kaspersky	montiera	norton	softonic	speedbit	sweetim	trendmicro	zonealarm
**asktoolbar**		.14	.05	.21	.03	.04	.18	.04	.10	.06	.05	.00	.06	.05	.24	.11	.01	.00
**avg**	.14		.01	.14	.03	.04	.13	.04	.09	.05	.02	.00	.04	.05	.13	.10	.01	.01
**avira**	.05	.01		.01	.01	.00	.01	.01	.01	.01	.00	.00	.00	.01	.01	.01	.00	.00
**babylon**	.21	.14	.01		.04	.04	.17	.05	.15	.07	.04	.00	.07	.07	.18	.16	.01	.01
**blekko**	.03	.03	.01	.04		.03	.03	.03	.04	.03	.02	.01	.02	.05	.02	.04	.01	.01
**conduit**	.04	.04	.00	.04	.03		.05	.04	.04	.03	.02	.00	.03	.08	.03	.05	.01	.00
**google toolbar**	.18	.13	.01	.17	.03	.05		.04	.09	.06	.05	.00	.09	.05	.18	.10	.02	.01
**imesh**	.04	.04	.01	.05	.03	.04	.04		.05	.03	.02	.00	.03	.04	.03	.05	.01	.01
**incredimail**	.10	.09	.01	.15	.04	.04	.09	.05		.06	.04	.01	.05	.06	.08	.12	.01	.01
**hotspot**	.06	.05	.01	.07	.03	.03	.06	.03	.06		.03	.00	.03	.04	.06	.06	.01	.01
**kaspersky**	.05	.02	.00	.04	.02	.02	.05	.02	.04	.03		.00	.00	.03	.05	.04	.00	.00
**montiera**	.00	.00	.00	.00	.01	.00	.00	.00	.01	.00	.00		.00	.00	.00	.00	.00	.00
**norton**	.06	.04	.00	.07	.02	.03	.09	.03	.05	.03	.00	.00		.03	.05	.05	.00	.00
**softonic**	.05	.05	.01	.07	.05	.08	.05	.04	.06	.04	.03	.00	.03		.04	.08	.01	.01
**speedbit**	.24	.13	.01	.18	.02	.03	.18	.03	.08	.06	.05	.00	.05	.04		.09	.01	.00
**sweetim**	.11	.10	.01	.16	.04	.05	.10	.05	.12	.06	.04	.00	.05	.08	.09		.01	.01
**trendmicro**	.01	.01	.00	.01	.01	.01	.02	.01	.01	.01	.00	.00	.00	.01	.01	.01		.00
**zonealarm**	.00	.01	.00	.01	.01	.00	.01	.01	.01	.01	.00	.00	.00	.01	.00	.01	.00	

### Identifying symbiotic and clash relationships via personalized pagerank

We argue that an alternative and potentially better method to identify symbiotic relationships is to apply a graph-based measure of *relative importance* using personalized PageRank. As in RQ1, if companies are in a symbiosis or a clash, then the relationships among their addons should reveal that. Provided with a query that consists of all addons of a company, PPR should increase or decrease scores of other companies’ addons as compared to their non-personalized PR scores. A substantial increase in the scores of a company’s addons should indicate its symbiosis with the query company, a marked decrease might tell that of a clash between them.

The general “importance” of company *c* is estimated by its *expected* PR score, which is the weighted sum of PR scores of the company’s addons. Denote as *s*_*i*_ the PR score of addon *a*_*i*_ that belongs to the set **A**_*c*_ of all addons of *c*. The expected score *S*_*c*_ (of the company *c*) will then be:

$$\begin{array}{c} S_{c}=\sum_{a_{i}\in A_{c}}p_{i}s_{i} \end{array}$$ (4)

where *p*_*i*_ is the probability of drawing addon *a*_*i*_ from all *c*’s addons. Specifically, *p*_*i*_ = *freq*(*a*_*i*_)/*freq*(**A**_*c*_), where *freq*(*a*_*i*_) is the frequency of addon *a*_*i*_ in terms of the number of user browsers in which *a*_*i*_ is installed; *freq*(**A**_*c*_) is the sum of frequencies of all *c*’s addons.

Similarly, for each company *c*_*i*_ in the PPR query, the algorithm computed the expected PPR score of every other company *c*_*j*_, and compared those scores with the original, non-personalized expected PR scores. Table 5 shows the *relative importance*: the ratio between the expected PPR score of company *c*_*j*_ given a query company *c*_*i*_ and the expected non-personalized PR score of *c*_*j*_. Red cells (low ratios) suggest a clash between the companies, and green cells (high ratios) a symbiosis. The ratios in Table 5 are non-symmetric, i.e. the expected PPR score of company *c*_1_ can decrease when *c*_2_ is in the query, while the expected PPR score of *c*_2_ can increase when *c*_1_ is in the query. This may indicate a complex relationship between the two companies: *c*_1_ and *c*_2_ can sign a contract according to which *c*_1_ helps distributing addons of *c*_2_, however *c*_2_ does not have to help distributing addons of *c*_1_. Moreover, *c*_2_ may even end up removing *c*_1_’s addons.

**Table 5:** Ratios of expected PPR and expected PR scores of companies. Columns are Companies in PPR Queries.

	asktoolbar	avg	avira	babylon	blekko	conduit	google toolbar	imesh	incredimail	hotspot	kaspersky	montiera	norton	softonic	speedbit	sweetim	trendmicro	zonealarm
**asktoolbar**		.85	1.23	.8	.76	.74	.55	.6	.8	.87	.61	.85	.51	.91	.66	.55	.67	.78
**avg**	.57		.72	.72	.87	.86	.55	.62	.87	.88	.55	.96	.48	.86	.64	.6	.7	.9
**avira**	.87	.77		.72	.63	.53	.53	.54	.63	.84	.47	.78	.38	.64	.62	.49	.54	.56
**babylon**	.53	.8	.71		.71	.66	.53	.55	.77	.79	.66	.79	.54	.8	.64	.52	.72	.8
**blekko**	.57	1.06	.78	.77		.99	.56	.71	1	1.07	0.63	1.49	.57	1.09	.69	.73	.71	1.15
**conduit**	.53	.95	.95	1.64	.79		.64	.72	2.04	1.05	.61	.92	.56	2.58	.67	.72	.89	1.07
**google toolbar**	.56	.79	.73	.79	.8	.77		.59	.88	.91	.63	.8	.56	.9	.66	.56	.84	.84
**imesh**	.58	.87	1.73	.88	.91	.83	.55		.87	.99	.66	.93	.53	.94	.7	.6	.78	.85
**incredimail**	.52	.77	.7	.62	.68	.59	.48	.53		1.06	.53	1.05	.4	.7	.57	.49	.55	.64
**hotspot**	.47	.77	.69	.67	.93	2.71	.5	.58	.65		.58	.76	.44	.74	.66	.52	.58	.72
**kaspersky**	.52	.7	.64	.75	.63	.67	.53	.55	.76	.8		.78	.37	.82	.66	.51	.61	.6
**montiera**	.61	.96	.79	.75	.78	.71	.54	.72	1.11	.91	.61		.52	1.14	.69	.77	4.31	.71
**norton**	.56	.72	.64	.73	.99	.75	.55	.67	.95	.79	.46	.78		.73	.62	.57	.73	.72
**softonic**	.55	.95	.87	.85	.8	1.16	.56	.71	1.02	1.12	.62	1.4	.53		.67	.84	.65	.75
**speedbit**	.52	.83	1.09	1.3	.68	.67	.53	.55	.82	.86	.66	.78	.5	.8		.5	.68	.8
**sweetim**	.54	.9	.79	.82	.89	.91	.57	.67	1.02	.96	.63	1.12	.54	1.06	.65		.74	.78
**trendmicro**	.54	.7	.59	.68	.75	.61	.63	.56	.71	.73	.49	.76	.5	.69	.65	.51		.6
**zonealarm**	.51	1	.78	.64	.99	.95	.52	.62	.96	.89	.52	.85	.48	.81	.65	.56	1.03	

**Fig 8:**
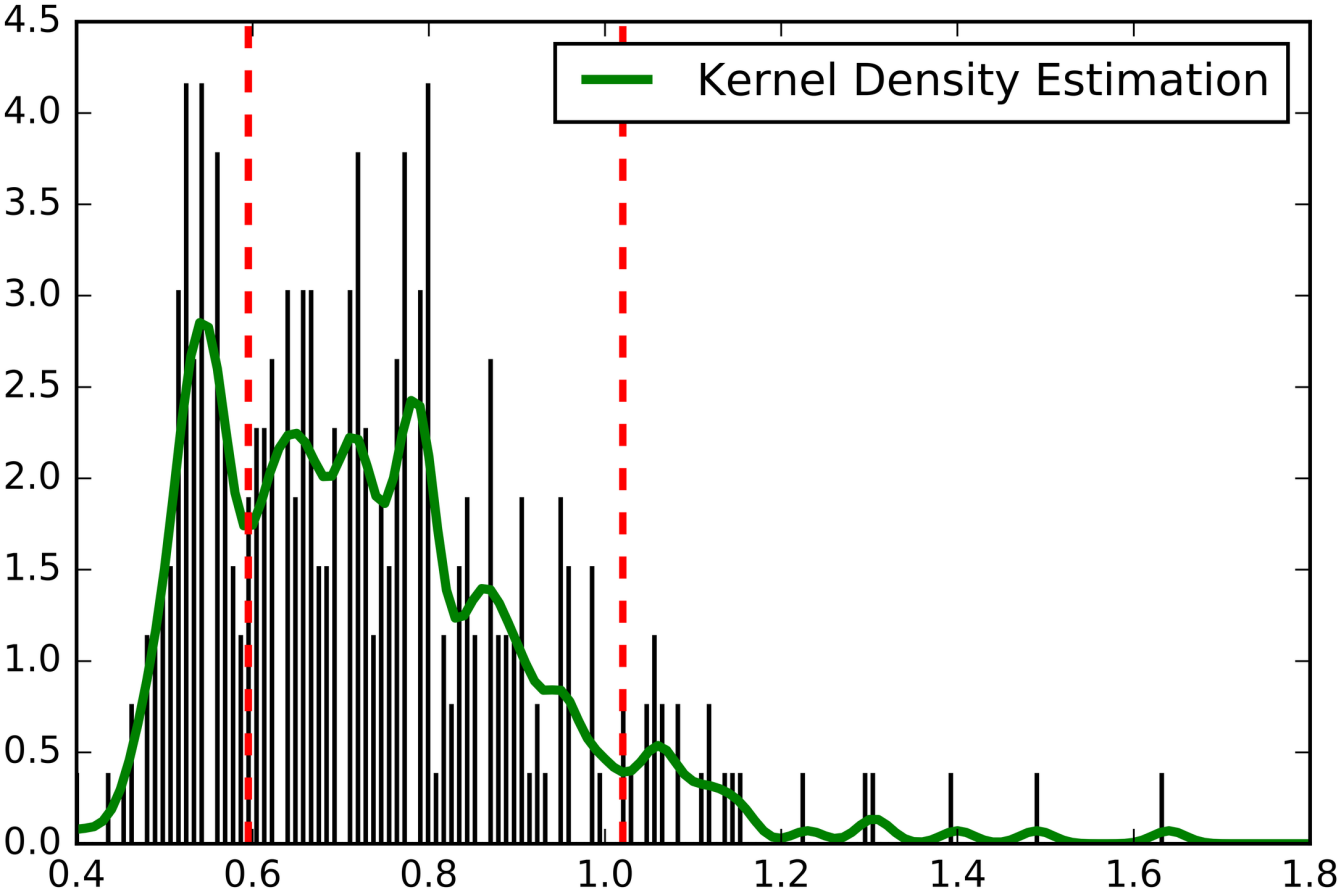
Kernel density estimation of the distribution of score ratios (see Table 5). Red vertical lines are score ratio cutoffs for clash (left) and symbiotic (right) relationships.

The red and green coloring in Table 5 is based on setting score ratio cutoffs at .6 for clashes and 1.02 for symbioses. The rationale behind setting those cutoffs is based on the results of a Kernel Density Estimation (Gaussian kernel, bandwidth = 0.1) performed on the distribution of values in Table 5. Fig 8 shows the Kernel Density Estimation output with the .6 and 1.02 cutoffs superimposed on it. The two cutoff values were determined by eyeballing the transition points in the Kernel Density Estimation graph. The range from .4 up to .6 resembles the beginning of a seemingly normal distribution, climbing to a peak and then declining. The range from .6 to 1.02 shows a considerably more gradual decline, with some wrinkles, suggesting that this is another strata in the values in Table 5. The range above 1.02 shows a straightening out of the graph. As there are no guideline on choosing the transition points in a Kernel Density Estimation graph, alternative ranges, suggesting other transition points, were also tried. Making the first transition point at .55 where the graph ends its initial incline and starts declining or making the second transition point more to the right of the 1.02 mark resulted in only minor changes in the coloring pattern in Table 5.

To verify the implied meaning behind the transition points in Fig 8, and the resulting coloring pattern in Table 5, a sample of the implied clashes and symbioses were examined. Market behavior seems to support the implied classification of symbioses and clashes. For example, the lowest implied symbiotic score ratio at 1.02 refers to the ratio of *Softonic* in *IncrediMail*’s PPR. A symbiosis could be expected between these companies. Because *Softonic* develops email addons, *IncrediMail* might be *Softonic*’s distributor. Indeed, *Softonic*’s “PostSmile works with the most popular email programs, including Outlook, Outlook Express, Eudora, Thunderbird, IncrediMail, AOL Mail and many others” [69]. However, even if *Softonic* was installed on a user’s machine, it could have arrived there through another email client—and so, *IncrediMail*’s score ratio is only 0.7 in Softonic’s PPR.

Another prominent example is the partnership between *IncrediMail* and *Conduit*. This symbiosis is well known (Conduit eventually acquired IncrediMail—now called Perion [70]). The score ratio of 2.04 of *Conduit* in *IncrediMail*’s PPR would suggest that if *IncrediMail* is installed, *Conduit*’s addons might be found as well. However, the opposite is not true. The coloring in Table 5 can also reveal less known symbioses. *Conduit* and *Babylon* have long been considered competitors. However, the score ratio of 1.64 of *Conduit* in *Babylon*’s PPR lifts a curtain over a possibly well-hidden agreement between the two companies. Indeed, *Conduit*’s and *Babylon*’s toolbars tend to appear together [71].

Another revealing result in the coloring of Table 5 is the implied relationship between *Avira Antivirus* and *ASK*. A collaboration between *Avira Antivirus* and the toolbar distributor *ASK* appears counterintuitive. A toolbar company is unlikely to promote the addons of an antivirus company considering that antiviruse software often treats toolbars as spyware. Nevertheless, *ASK*’s score ratio in *Avira Antivirus*’s PPR is 1.23. This suggests that when *Avira* *Antivirus* is installed there is a higher probability of *ASK* addons being found. Indeed, there is a market symbiosis between these two companies. *Avira Antivirus*’s official website states that “Avira chose Ask.com to be our partner in bringing you the SearchFree Toolbar” [72]. Likewise, the high score ratio of *iMesh* in *Avira Antivirus*’s PPR is intriguing. Here too, a discussion on the official *Avira* website might hint about a connection between the two companies [73].

Score ratios below 0.6 may indicate clashes between competing companies. It is known that the addon space is very competitive and that many clashes occur. For example, the majority of antivirus products clash with each other. Since in most cases two antivirus software products cannot coexist on the same computer, when one product is installed, the other often gets uninstalled. Likewise, antivirus software tends to remove toolbars and other addons. In Table 5, columns corresponding to major antivirus companies, such as *Kaspersky* and *Norton*, contain many red cells. This implies that wherever *Kaspersky* or *Norton* is installed, other antiviruses and toolbars are rarely seen. Interestingly, smaller antivirus companies, such as *Avira* and *TrendMicro*, have many red cells in corresponding rows. It is remarkable that the free antivirus tool, *AVG*, appears to live in harmony with other companies without seeming to remove toolbars or browser addons [74]. In the toolbar domain, *ASK* and *Google* appear to be the largest offenders. As discussed at the beginning of this section, *ASK* is known for removing millions of rival toolbars, while “Google toolbar … prevents other toolbars being installed into your computer” [75].

## Discussion

### Summary of results

The ecosystem of the Web browser is a mostly unexplored research area. This research is the first to the best of our knowledge to measure this dynamic environment with its important economic and security consequences. Its importance is highlighted by the observation that addon manufacturers engage in partnerships and compete with each other by supporting and suppressing the distribution of each other’s addons. As most of this dynamics is hidden from the eyes of ordinary Web users, this activity also has serious privacy issues involved. Being able to measure these activities can open the door to at least partially monitoring these activities and potentially alleviate some of the privacy issues. Accordingly, the goal of this study was to develop tools and methodologies for measuring activity in this complex ecosystem.

The study, analyzing a unique dataset of addons installed on almost a million machines, applied Personalized PageRank (PPR) to capture relationships between vertexes in the graph. The results show that the Web browser ecosystem can be tested to identify removed addons. Armed with this observation (RQ1) and with the methodology developed to test RQ2, the results show that symbiosis and clashes can be identified, and better so with PPR than with a non-personalized method. The results show that some companies are engaged in symbiotic relations—when one company’s addons are installed on a machine, there is a good chance that another company’s addons will be there too. Other companies clash with each other in the addon ecosystem—seemingly removing the addons of specific other companies.

### Direct implications

The ability to measure the extent that addons tend to appear or not to appear together suggests a method to actively detect symbioses and clashes between addon distributing companies. This could have important manifestations for regulators and for addon companies. Information about a symbiosis between two companies can help better analyze the powers and driving forces in the addon ecosystem, and so allow competitors to better prepare and regulators to better regulate it. Companies placing addons could benefit by knowing in reality which other companies support them and which oppose them by monitoring the actual way in which addons apparently suppress or distribute their addons. Importantly, regulators could gleam insight into actual addon ecosystem behavior to identify economic oligarchies, often regulated in other economic environments, and so ensure more open competition. And, from a user perspective, regardless of the legality of removing or adding new addons without user approval, being able to monitor these addon clashes and symbioses could go a long way towards building a trustworthy and open addon ecosystem.

Moreover, while it is unlikely that a company might not be aware of a symbiosis between its own addons and addons of another company, information about a clash that involves the company’s addons may sometimes come unexpectedly. A typical addon manufacturer can benefit from the information about a clash between their own and someone else’s addons in many ways, including that:

1. Such a clash may imply that the user prefers another company’s addon over their own addon, so there might be a way to perform a comparative analysis of the two addons and learn how to improve their value proposition.
2. The clash could mean that a newly installed addon is hostile to other addons in a potentially illegal way, i.e. it is the addon—and not the user—that uninstalls or sabotages another addon. If so, the distributor of the removed addon could report an abuse.
3. An addon manufacturer can ask a third-party distributing company not to install an addon on a machine that keeps the hostile addon. Since in many cases addon developers pay distributors per install, this could decrease the developers’ costs and raise their profits in the long run.
4. A clash can occur between addons of seemingly non-competing companies. This may happen when something goes wrong in the distribution process and the problem slips off the company’s radar. In this case, precise information about unexpected clashes might help the affected company quickly fix the problem.
5. The addon ecosystem may be so complex that distribution monitoring is barely possible. If an unintended clash is detected, the owner of the affected addon can contact the owner of the hostile addon and ask them to act.

### Broader implications and agency relationships

On a broader perspective, measurably implied clashes and symbioses in the addon ecosystem might suggest that lessons learnt from other kinds of economic ecosystems might apply to the addon ecosystem too. A perhaps pertinent example of this is Agency Theory [76]. Agency theory deals with contractual relationships between principals and agents who might be individuals or company representatives. The agency theory perspective is central to understanding when and how people and companies contract with each other [77]. In agency theory, a principal lets out work to an agent in a context of information asymmetry characterized by the agent knowing more about its own capabilities and actions than the principal can possibly know. This opens up the principal (who in the case of the addon ecosystem is the user allowing companies to install addons on his/her machine) to several risk categories from the agents, who in this case are the companies installing those addons. These risks are classified in agency theory into three broad categories widely known as adverse selection risks, moral hazard risks, and unforeseen contingencies. Adverse selection risks are risks associated with not knowing enough about the agents competing on the contract before awarding it to one of them. Often, principals are not fully aware of the capabilities or track record of the competing agents when choosing among them. This allows agents to oversell their capabilities and to masquerade as something they are not. Moral hazard risks are risks associated with the actions of the agent who has been awarded the contract to do the work. Typically, principals are not capable or do not have the resources to carefully oversee everything an agent is doing for them. This allows the agent to take advantage of the principal without the principal being aware of it. Unforeseen contingencies refer to the cost of dealing with unexpected events that were not included in the contract.

In the case of an addon ecosystem, adverse selection risks deal with users not fully knowing the consequences of their granting permission to addon companies to install addons and their inability to investigate the capabilities of those companies and what they are really after. The fact that users may not even realize that they should monitor the addon companies before granting them permission, let alone even being able to know how to do so, increases the magnitude of such adverse selection risks. Moral hazard risks in an addon ecosystem may entail precisely the kind of hidden symbioses and clashes investigated in this study. Specifically, it would seem that addon companies are adding and removing other addons without the knowledge or consent of the user. Activities concerning the principal that are done without his/her knowledge or consent are typical of moral hazard risks. Importantly, in this context, at least some adverse selection and moral hazard risks can be somewhat alleviated by the principal being able to—or, as often happens in the real world, by a regulating agency or by other competing agents being able to—measure the behavior of the agents. Knowing of preexisting clashes and symbioses in the addon ecosystem could inform users of the possibility that the addons may do more than the user expects them to do. Knowing of such relationships through monitoring the ecosystem, as shown in RQ2, could at least partly inform the principals of the potential for such a risk. As RQ1 shows, knowing that such an activity actually occurred, moral hazard can also be identified.

Applying the agency theory perspective may allow the transformation of the addon ecosystem into a mature marketplace. Within the agency theory context, the ability to control—or at least to measure—some of the adverse selection and moral hazard categories of risk is a key determinant of the price of the contract and whether the contract will be a fixed price or a time and materials one [78]. Applying agency theory to the context of addons might suggest that being able to measure the way addons interact with each other, i.e. measure agent behavior in removing and installing other addons, may affect the pricing of those services and, once a market for such measurements becomes viable, also the very nature of the contracting. Even if users cannot be expected to apply the type of algorithms shown in this paper, regulators and competing companies can be expected to. Regulators can be expected to take action if competition in the market is reduced. Competing companies can be expected to take action if their profit margins or access to information about potential clients will be affected by having their own addons removed.

Once such algorithms are applied and such agency risks identified, as in other agency theory contexts, users could demand discounts or rebates for allowing addons to be installed or removed from their machines. This is much as customers currently expect from using their loyalty cards that allow stores to track their purchase activities. As with loyalty cards, users could demand discounts or rebates because their privacy exposure is increased by those activities. The industry already has a well-established market for paying other websites to direct traffic their way. Users could demand a cut of that profit as compensation for being tracked. Likewise, users could demand bonuses or rebates because of their increased exposure to a larger pool of addon companies through the automatic installing of addons by companies in symbioses with each other. Being able to measure such activity, as shown in this paper, is the first step towards making such a transformation.

### Conclusion

The methodology proposed in this study investigates a previously unexplored domain of Web browsers and the ecosystem of their addons. The results show that in the Web browser ecosystem addons have symbiotic and clash relationships. The process described in this paper could allow a method to detect, and in doing so also regulate, this ecosystem with limited manual intervention. This could transform the current unwieldy addon ecosystem to a more traditional agency type market.
